# Inhibition of prostaglandin E2 receptor 4 by lnc000908 to promote the endothelial‐mesenchymal transition participation in cardiac remodelling

**DOI:** 10.1111/jcmm.14524

**Published:** 2019-07-12

**Authors:** Xingxing Chen, Wenhua Ge, Jie Hu, Tiancheng Dong, Hui Yao, Lingzhi Chen, Bin Geng, Hao Zhou

**Affiliations:** ^1^ Department of Cardiology The First Affiliated Hospital of Wenzhou Medical University Wenzhou China; ^2^ Stomatological Hospital, College of Medicine Xi'an Jiaotong University Xi'an China; ^3^ Department of Clinical Laboratory Wenzhou Central Hospital Wenzhou China; ^4^ Hypertension Center of Fuwai Hospital, State Key Laboratory of Cardiovascular Disease, National Center for Cardiovascular Diseases Chinese Academy of Medical Sciences and Peking Union Medical College Beijing China

**Keywords:** cardiac fibrosis, endothelial‐mesenchymal transition, long non‐coding RNAs, prostaglandin E2 receptor 4

## Abstract

Long non‐coding RNAs (lncRNAs) have emerged as potent regulators of cardiac disease; however, the role of lncRNA in cardiac fibrosis remains partially understood. In this study, we identified a cardiac endothelial‐enriched lncRNA‐lnc000908, which was markedly up‐regulated in rats with cardiac fibrosis. In addition, the expression of prostaglandin E2 receptor 4 (EP4) was decreased in cardiac fibrosis. In vivo lnc000908 silencing by lentivirus increased the EP4 level, decreased endothelial‐mesenchymal transition (EndMT) and improved cardiac fibrosis and cardiac function. Consistently, the lnc000908 knockdown also up‐regulated EP4 and suppressed transforming growth factor‐beta (TGF‐β)‐induced EndMT in cardiac microvascular endothelial cells. In contrast, the lnc000908 overexpression by lentivirus decreased the EP4 level and induced EndMT. Of note, these pro‐ or anti‐EndMT effects were reversed by the EP4 overexpression or the EP4 antagonist AH‐23848, respectively. This study demonstrates that lnc000908 is a novel regulator of cardiac fibrosis by modulating the EP4 expression and EndMT.

## 1 INTRODUCTION

Cardiac fibrosis, characterized by the excessive accumulation of fibroblasts and extracellular matrix, is a common pathological manifestation in the late stage of various cardiovascular diseases and results in the increased occurrence of sudden cardiac death, heart failure and arrhythmia.^1^, ^2^ Besides the resident collagen‐producing cardiac fibroblasts (CFs),^1^ fibroblast‐like cells derived from endothelial cells also contribute to the pathogenesis of cardiac fibrosis by the endothelial‐mesenchymal transition (EndMT; endothelial cells lose its morphological characteristics, endothelial markers such as cluster of differentiation‐31 [CD31], and display a mesenchymal phenotype).^3^ In the fibrotic heart of patients with chronic kidney disease, around 17% of fibroblasts are derived from EndMT.^4^ Furthermore, EndMT has been reported to contribute to cardiac fibrosis in endothelin‐1 knockout diabetic mice^5^ and isoproterenol (Iso)‐induced heart failure rats.^6^, ^7^ The patients' and animal models' evidence suggests that EndMT plays a vital role in cardiac remodelling.

Prostaglandin E2 receptor 4 (EP4), also known as Ptger4, is a prostaglandin E2 (PGE2) receptor subtype,^8^ which is reportedly involved in the development of various cardiovascular diseases such as myocardial hypertrophy, myocardial ischaemia‐reperfusion injury, myocarditis, heart failure and atherosclerosis.^9^, ^10^ Reportedly, EP4 silencing increases the collagen content and aggravates cardiac fibrosis^11^; in contrast, activation of EP4 by ONO‐0260164 reduces collagen deposition.^12^ In some studies, EndMT was inhibited by PGE2 stimulation^13^ and then rescued by selective EP4 antagonists AH‐23848.^14^ A recent study reported that the EP4 agonist, L‐902688, also suppressed EndMT and then attenuated right ventricular cardiac fibrosis in pulmonary arterial hypertension rats.^15^ These studies highlight that the EP4 activation reduces cardiac fibrosis by inhibiting EndMT.

Long non‐coding RNAs (lncRNAs) are a class of non‐coding RNA with a length larger than 200 nucleotides; some of them participated in the pathogenesis of heart development,^16^ hypertrophy,^17^ heart failure,^18^ myocardial infarction^19^ and cardiac fibrosis.^20^ Recently, lncRNA MALAT1 modulated TGF‐β1‐induced EndMT by the down‐regulation of miR‐145 in neointimal hyperplasia.^21^ The overexpression of lncRNA uc.77 and 2700086A05Rik induced epithelial‐mesenchymal transition (EMT, a process similar to EndMT) in human lung epithelial cells and might facilitate the development of pulmonary fibrosis.^22^ These data indicate that lncRNAs might mediate EndMT contribution to cardiac fibrosis. However, whether some lncRNAs target EP4 receptor to regulate EndMT remains unclear.

This study aims to perform the RNA sequencing in a rat model of heart fibrosis and identify a cardiac endothelial‐enriched lncRNA‐lnc000908, which is markedly up‐regulated in rats with cardiac fibrosis. The lnc000908 gene located in the upstream of the EP4 receptor gene and the overexpression of lnc000908 down‐regulated the EP4 expression. By lentivirus‐delivery shRNA to the knockdown of lnc000908 in the heart enhanced the cardiac pump function and inhibited cardiac fibrosis by increasing EP4 to lower EndMT.

## 2 MATERIALS AND METHODS

### 2.1 Cardiac fibrosis model

This study was approved by the Animal Ethics Committee of Wenzhou Medical University and was consistent with the National Institutes of Health Guide for the Care and Use of Laboratory Animals. We obtained Sprague Dawley rats (age: 6 weeks, male, 200‐220 g) from the Shanghai Experimental Animal Center. A cardiac fibrosis model was induced by subcutaneous injection of Iso (5 mg/kg/d) for 7 consecutive days and then kept for another 14 days until the endpoint. Similarly, the controls were administered an equal volume of saline. We harvested hearts that were stored in RNA later™ Stabilization Solution (Ambion) for lncRNA analysis.

### 2.2 RNA sequencing

We performed RNA sequencing on fibrotic and control hearts (3 samples each group) with service from Novogene per the manufacturer's standard protocols. Briefly, 3 μg of RNA per sample was used as input material for the RNA sample preparations. In addition, sequencing libraries were generated using the rRNA‐depleted RNA by NEB Next^®^ Ultra™ Directional RNA Library Prep Kit from Illumina^®^ (NEB). After cluster generation, the libraries were sequenced on an Illumina HiSeq 2500 platform, and 125‐bp paired‐end reads were generated. Transcripts with *P*_adjust_ < 0.05 were assigned as differentially expressed. Furthermore, the Gene Ontology (GO) and KEGG (Kyoto Encyclopedia of Genes and Genomes) pathway analysis were performed to ascertain the role of the closest genes to which lncRNAs are preferentially located.

### 2.3 **Lentiviral vector construction and animals**'** in vivo study**

In this study, we used lentivirus (Hanbio Inc) to conduct the loss‐of‐function experiment. Lnc000908 was amplified by PCR (polymerase chain reaction) and cloned into a backbone plasmid (lenti‐sh908). The negative control plasmid (lenti‐ctrl) was lack of lnc000908 sequence. Lentivirus (2 × 10^9^ pfu/rat) was administered in one into the tail vein before Iso infusion. The lenti‐ctrl infused group served as negative controls. After 21 days, rats were killed with an overdose of pentobarbital (100 mg/kg, one dose intraperitoneally). The hearts were harvested for future analysis. We randomly divided 6‐week‐old male Sprague Dawley rats into the following four groups: (a) control; (b) cardiac fibrosis model, Iso treatment; (c) Iso + lenti‐ctrl; and (d) Iso + lenti‐sh908. Target sequences of shRNA, 5′‐GCAGATAGCATCAAGTTGATGAGATT‐3′.

### 2.4 Echocardiograph

We performed transthoracic echocardiography using a Sonos 5500 ultrasound machine (Phillips) with a 12‐MHz probe on days 21, as described previously.^23^ Two‐dimensional, M‐mode, Doppler images were obtained in the parasternal long‐axis view. The left ventricular end‐systolic and end‐diastolic diameters (LVESd, LVEDd) were measured in three non‐repeating images and averaged. Then, we evaluated left ventricular ejection fraction (EF%) and fractional shortening (FS%).

### 2.5 **Haematoxylin and eosin and Masson**'**s trichrome staining**

Hearts samples were fixed in 4% formalin overnight, embedded in paraffin and sectioned; we processed 4‐μm sections for haematoxylin and eosin (H&E) staining using standard histological procedures to assess the general histological appearance. In addition, the routine Masson's trichrome staining (GenMed Scientifics Inc) was performed to observe the collagen deposition (blue). We examined the sections under an optical microscope (Nikon Corp.) and measured collagen areas using Image‐Pro Plus (Media Cybernetics, Inc.).

### 2.6 Enzyme‐linked immunosorbent assay for the collagen content

Heart tissues were weighed and sliced into pieces on ice, then homogenized (100 mg tissue per mL of ice‐cold homogenizer buffer) and centrifuged at 15 000 g for 20 minutes at 4°C. The supernatant was collected to measure the content of type I and III collagen with enzyme‐linked immunosorbent assay (ELISA) Kits (Boyun Biotech) as per the manufacturer's instructions. The experiment was repeated three times at least.

### 2.7 Quantitative real‐time PCR

We extracted the total RNA with TRIzol (Invitrogen), and 2 μg of RNA was used for the cDNA synthesis by reverse transcription. The real‐time PCR was performed on the LightCycler^®^96 Real‐Time PCR System (Roche) using the SYBR green method. The PCR conditions comprised a denaturation step at 95°C for 30 seconds, then 40 cycles of amplification at 95°C for 10 seconds and 60°C for 10 seconds. Next, lncRNA expression levels were normalized to GAPDH. The relative expression of the lncRNAs was assessed using the 2^−∆∆^CT method. All primer sequences used were listed as follows: lnc000908, forward 5′‐AGGGAGGAGGGTGGTAGT‐3′ and reverse 5′‐GGTTTGTTTGTGAGGTGTTT‐3′; EP4, forward 5′‐ TCGCGCAAGGAGCAGAAGGACAC‐3′ and reverse 5′‐ GACGGTGGCGAGAATGAGGAAGGA‐3′.

### 2.8 Western blot analysis

We extracted proteins from heart tissues and cultured cells using lysis buffer and centrifuged at 10 400 g for 10 minutes at 4°C. Protein concentrations were quantified by the BCA method (PC0020; Solarbio Life Sciences). Next, 80 μg samples were added on the gel for SDS‐PAGE and then transferred to PVDF membranes. After blocking with 5% non‐fat milk for 1 hour at room temperature, membranes were incubated with primary antibodies overnight at 4°C and then with horseradish‐conjugated secondary antibodies. The protein expression was quantified by the Bio‐Rad Gel Image Analysis System (Bio‐Rad) and Image‐Pro Plus (Media Cybernetics, Inc). The total protein levels were normalized to GAPDH. The primary antibodies used were as follows: anti‐CD31, ab28364; anti‐VE‐cadherin, ab33168; anti‐α‐SMA, ab5694; anti‐Vimentin, ab92547, all from Abcam, 1:500; anti‐EP4, 24985‐1‐AP, Proteintech, 1:500; and rabbit anti‐GAPDH.

### 2.9 Cell culture

We purchased primary rat cardiac microvascular endothelial cells (CMECs), cardiac fibroblasts (CFs), cardiomyocytes (CMs) from the cell bank of the Chinese Academy of Science. Cells were cultured in Dulbecco's modified Eagle's medium (DMEM; Lonza) with 10% foetal bovine serum (FBS; Gibco), 100 U/mL penicillin (Gibco) and 100 mg/mL streptomycin (Gibco). All cells were maintained at 37°C in 5% CO_2_ incubator.

### 2.10 Lentivirus construction, cell transfection and treatment

We purchased recombinant lentivirus targeting lnc000908 or EP4 from Hanbio Co., Ltd.. For the knockdown of lnc000908, cells were transfected with non‐target control siRNA (si‐ctrl) or lnc000908‐specific siRNA (si‐908) using Lipofectamine 2000 (Cat:11668027; Invitrogen) as per the manufacturer's protocol. For the overexpression study, cells were infected with lnc000908‐targeted lentivirus (LV‐908) or non‐target control lentivirus (LV‐ctrl). Cells were randomly grouped according to different treatment (n = 4) plans as follows: (a) control; (b) TGF‐β; (c) TGF‐β + si‐ctrl; (d) TGF‐β + si‐908; (e) TGF‐β + si‐908 + AH‐23848; (f) LV‐ctrl; (g) LV‐908; and (h) LV‐908 + LV‐EP4. After 48‐hour transfection, cells were exposed to TGF‐β (10 ng/mL) for another 24 hours. Selective EP4 antagonist AH‐23848 (10 μM) was administrated 4 hours after TGF‐β exposure. Primary CMECs were pre‐incubated with LV‐908 for 48 hours and further infected with LV‐EP4 for additional 48 hours.

### 2.11 Immunofluorescence assay

We performed immunofluorescence staining of vascular endothelial‐cadherin (VE‐cadherin), CD31, vimentin and α‐smooth muscle actin (α‐SMA). Cells were seeded in chamber slides, fixed with 4% formaldehyde and then blocked with 0.1% Triton X‐100 (Sigma‐Aldrich). Next, sections or slides were incubated with primary antibodies for α‐SMA (A5228; Sigma), vimentin (ab92547; Abcam), VE‐cadherin (ab33168; Abcam) or CD31 (ab28364; Abcam) for overnight at 4°C. Then, slides were washed and stained with fluorophore‐conjugated secondary antibodies for 2 hours at room temperature. The results were analysed by fluorescence microscopy (Nikon Corp.) and processed with Image‐Pro Plus (Media Cybernetics). Negative controls were performed by incubating cells or tissues with secondary antibodies in the absence of specific primary ones.

### 2.12 Fluorescence in situ hybridization (FISH) for lnc000908

Fibrotic (21 days after Iso treatment) and normal hearts were fixed in 4% formalin for 48 hours, and paraffin sections of 5 μm were sliced. Sections were dewaxed, dehydrated through a series of diminishing concentrations of ethanol, dried in a microwave oven for 25 minutes and cooled down to room temperature. After washing three times in 1× PBS, sections were incubated for 5 minutes in 1‐mL ice‐cold PBS containing 0.5% Triton X‐100 at 4°C. Subsequently, sections were rinsed three times for 5 minutes with 1× PBS and blocked with 100 μL of the pre‐hybridization buffer (a mixture of 100× blocking solution and 1× pre‐hybridization buffer) at 37°C for 30 minutes. Under dark conditions, mixed 1× hybridization buffer with either 2.5 μL of 20 μM lnc000908 FISH Probe Mix or control FISH Probe Mix into solution, which was then added to sections for hybridization overnight at 37°C. The next day, we washed the slices once with 4× SSC, 2× SSC, 1× SSC in order at 42°C and with 1× PBS for 5 minutes. Finally, sections were stained with DAPI. The localization of lnc000908 in the myocardial tissue was observed under the fluorescence microscope (Nikon Corp.) at different magnifications. The Fluorescent In Situ Hybridization Kit and Cy3‐labelled probe were purchased from RiboBio Co., Ltd.

### 2.13 Statistical analysis

Data are expressed as the mean ± SD. All statistical analyses were performed with SPSS17.0 software. We used one‐way ANOVA for multigroup comparison (>2 groups) and the Student's *t *test for two‐group comparison. Furthermore, we considered *P* < 0.05 as statistically significant.

## 3 RESULTS

### 3.1 Identification of a cardiac microvascular endothelial‐enriched lncRNA that was up‐regulated in cardiac fibrosis

**Figure 1:**
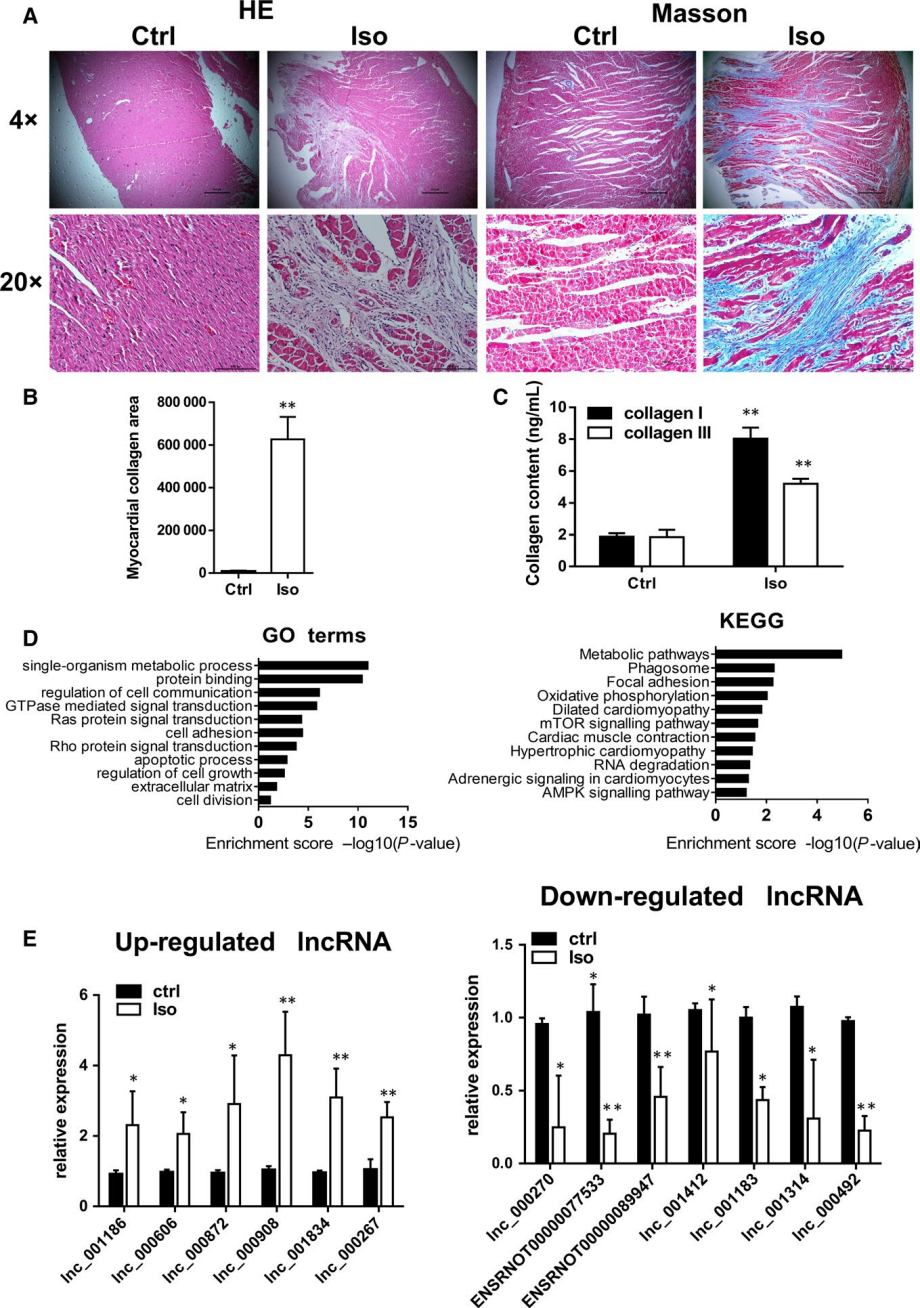
Expression profiles of lncRNAs in cardiac fibrosis. A‐C, The successful establishment of cardiac fibrosis in rats induced by isoproterenol was confirmed by HE & Masson's trichrome staining (A), myocardial collagen areas (B) and ELISA analysis for collagen I and III (C). Upper panel: magnification ×4, lower panel: magnification ×20, n = 5‐7. D, GO and KEGG pathway analysis of differentially expressed lncRNAs. E, Differential expression of representative lncRNAs was verified by qRT‐PCR on day 21. n = 3‐5 per group. Each experiment was repeated at least for three times. Data are presented as means ± SD. *P < 0.05, **P < 0.01 vs ctrl; ctrl, control; Iso, isoproterenol

First, we made the cardiac fibrosis rat's model by injecting Iso for 1 week. After another 2 weeks, rats were killed, heart tissues (about 100 mg) were removed for total RNA extraction, and remnant tissue for preparing a pathological slice. Haematoxylin and eosin and Masson's trichrome staining (Figure 1A) revealed that Iso treatment caused myocardial fibres disarrangement, leucocyte infiltration and massive proliferation of fibrous tissue. Furthermore, total collagen areas (blue area in Masson's stain, Figure 1B) and the ratio of collagen I to III (ELISA assay, Figure 1C) were increased in the heart, confirming the cardiac fibrosis in our animal model.

The RNA‐seq analysis identified 118 up‐regulated lncRNAs and 41 down‐regulated lncRNAs in the heart tissues of cardiac fibrosis. The GO analysis suggested that the enriched functional terms in lncRNAs included single organism, metabolic process, regulation of cell communication and cell adhesion (Figure 1D). The KEGG pathway analysis revealed that the top enriched pathways in lncRNAs included metabolic pathways, phagosome, focal adhesion and dilated cardiomyopathy (Figure 1D). With a cut‐off fold change of ≥2.0, we validated some lncRNA changes by quantitative real‐time PCR (qRT‐PCR) and confirmed the increased expression of lnc000908, lnc000872, lnc000606, lnc001834, lnc001186 and lnc000267; the expression of lnc000270, ENSRNOT00000089947, ENSRNOT00000077533, lnc000492, lnc001314, lnc001183 and lnc001412 decreased in the fibrotic heart (Figure 1E).

**Figure 2:**
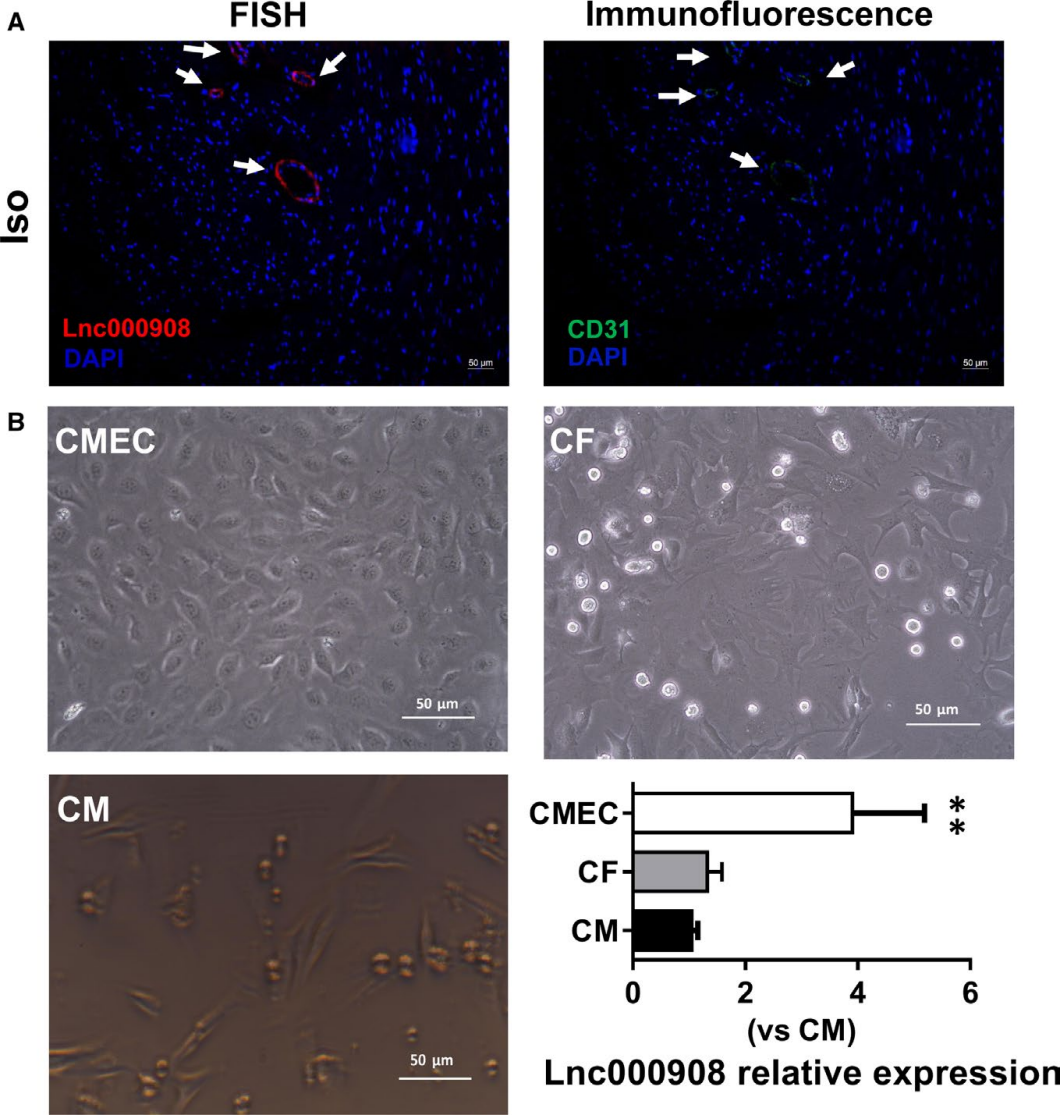
Lnc000908 enriches in cardiac microvascular endothelium. A, Fluorescence in situ hybridization (FISH) showed that lnc000908 (red) is dominantly expressed in the endothelium (CD31 immunofluorescence staining, green) of fibrotic heart (n = 3). Red, lnc000908; green, CD31; nucleus was stained blue with DAPI. B, QRT‐PCR analysis of lnc000908 expression in primary CMs, CFs and CMECs. Each experiment was repeated at least for three times. Data are presented as means ± SD. **P < 0.01 vs CM

Of these changed lncRNAs, the expression of lnc000908 was quite noticeable and increased about fourfold in the fibrotic heart. Remarkably, FISH image revealed that the lnc000908 (red colour) dominantly expressed in the endothelium (CD31 immunofluorescence staining, green colour) of fibrotic heart (Figure 2A); to validate it, we cultured primary CMECs, CFs and CMs. As shown in Figure 2B, qRT‐PCR revealed that the expression of lnc000908 in CMECs was the highest among the three kinds of cells. These findings suggested that lnc000908 is a CMEC‐enriched lncRNA.

### 3.2 Silencing lnc000908 reduced cardiac fibrosis by inhibiting EndMT

**Figure 3:**
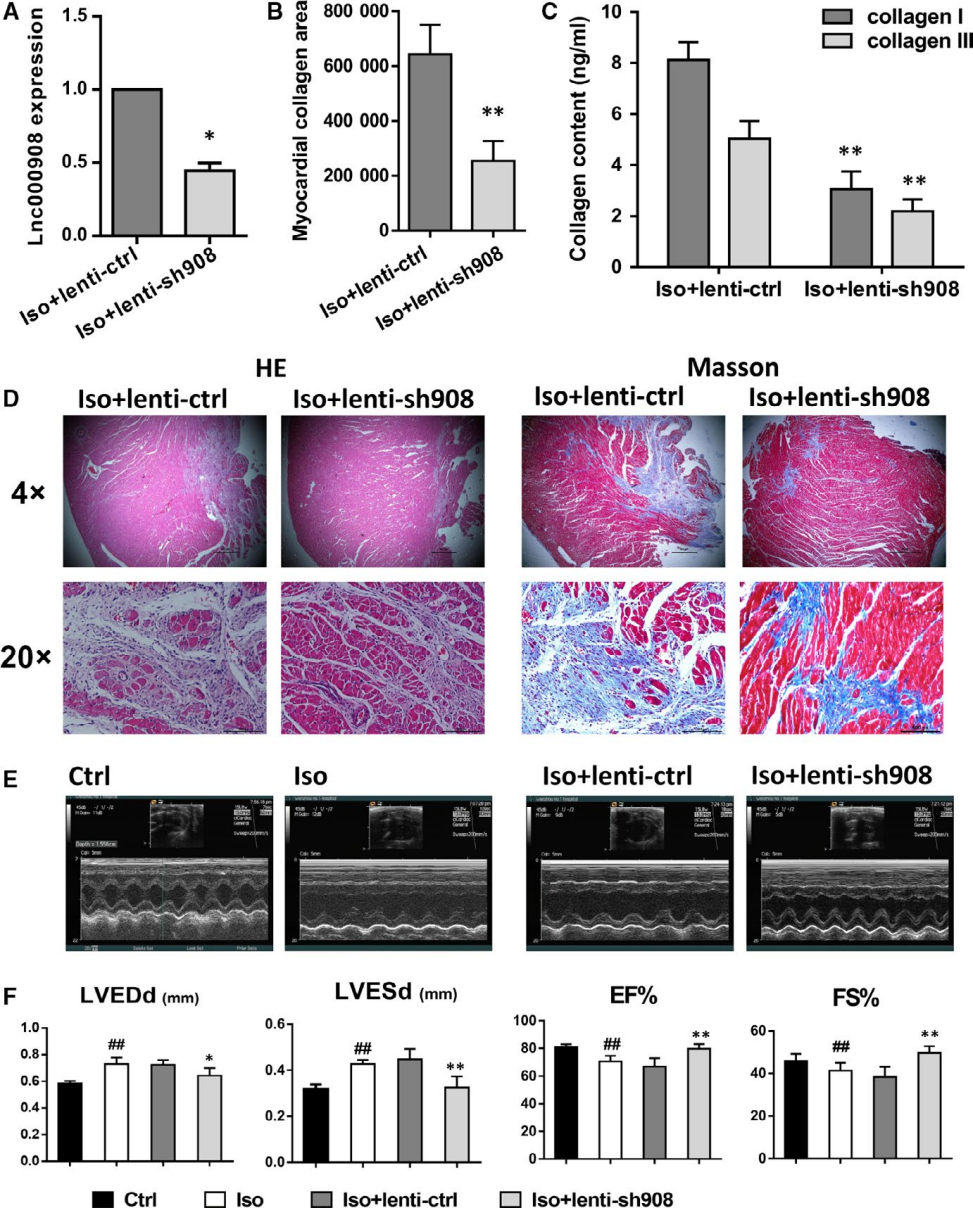
Knockdown of lnc000908 improves cardiac fibrosis and heart function. A, For knockdown, lentivirus (2 × 10^9^ pfu/rat) was administered by one dose injection to the tail vein before Iso infusion. The infection efficiency was assessed by qRT‐PCR analysis of lnc000908 expression, n = 4. B, Quantification of myocardial collagen area, n = 7. C, ELISA analysis for collagen I and III in heart tissues, n = 6. D, Representative images of HE & Masson's trichrome staining. Upper panel: magnification ×4, lower panel: magnification ×20, n = 7. E, Representative M‐mode images of echocardiography on day 21 before killing, n = 4. F, Echocardiographic assessment of cardiac dimension (LVEDd, left ventricular end‐diastolic internal diameters; LVESd, left ventricular end‐systolic internal diameters) and cardiac pump function (EF%, ejection fraction; FS%, fractional shortening), n = 4. Each experiment was repeated at least for three times. Data are presented as means ± SD. ##*P* < 0.01 vs ctrl; *P < 0.05, **P < 0.01 vs Iso + lenti‐ctrl

To elucidate the potential role of lnc000908 in cardiac fibrosis, we delivered the shRNA by lentivirus (lenti‐U6‐shRNA‐GFP) through tail vein injection; the next day, all animals received Iso treatment for the cardiac fibrosis model. We assessed the lentiviral transfection efficiency by qRT‐PCR analysis, and the results revealed that the lnc000908 expression was down‐regulated by about 55% compared with the control virus (lenti‐U6‐scramble RNA‐GFP; Figure 3A). The heart H&E and Masson's staining revealed that silencing lnc000908 reduced CMs disarray, fibrous hyperplasia (Figure 3B,D), and the level of collagen I and III (Figure 3C) induced by Iso. Furthermore, correlation with amelioration of cardiac fibrosis, cardiac pump function (by echocardiography analysis on days 21; Figure 3E), such as LVESd, LVEDd, EF and FS (Figure 3F), also improved.

**Figure 4:**
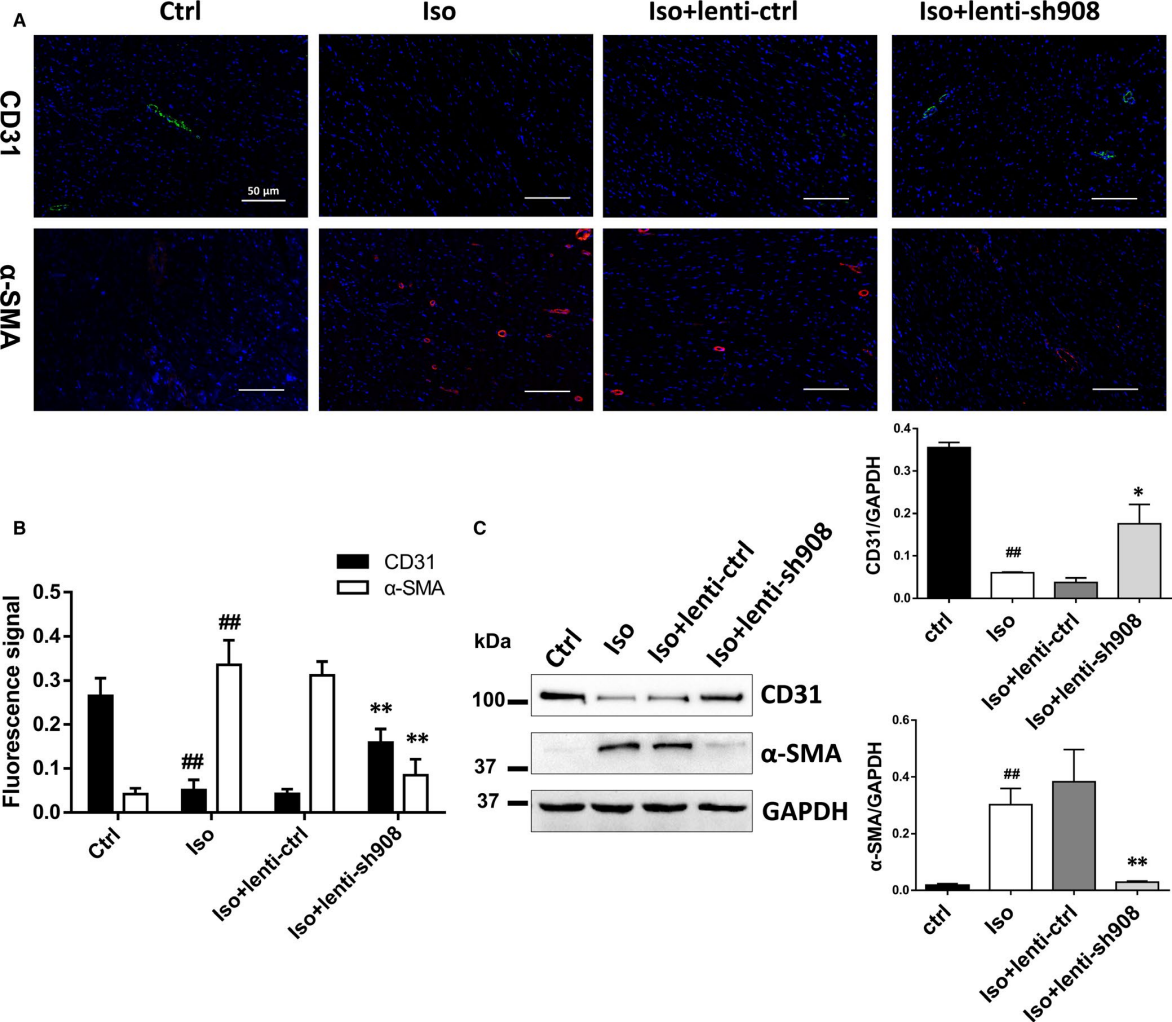
Lnc000908 knockdown attenuates cardiac EndMT in vivo. Immunofluorescence assay (A, B) and Western blot analysis (C) of α‐SMA and CD31 in heart tissues, n = 3‐4. Magnification ×20. Red, α‐SMA; green, CD31; nucleus were stained in blue with DAPI. Each experiment was repeated at least for three times. Data are presented as means ± SD. ##*P* < 0.01 vs ctrl; *P < 0.05, **P < 0.01 vs Iso + lenti‐ctrl

**Figure 5:**
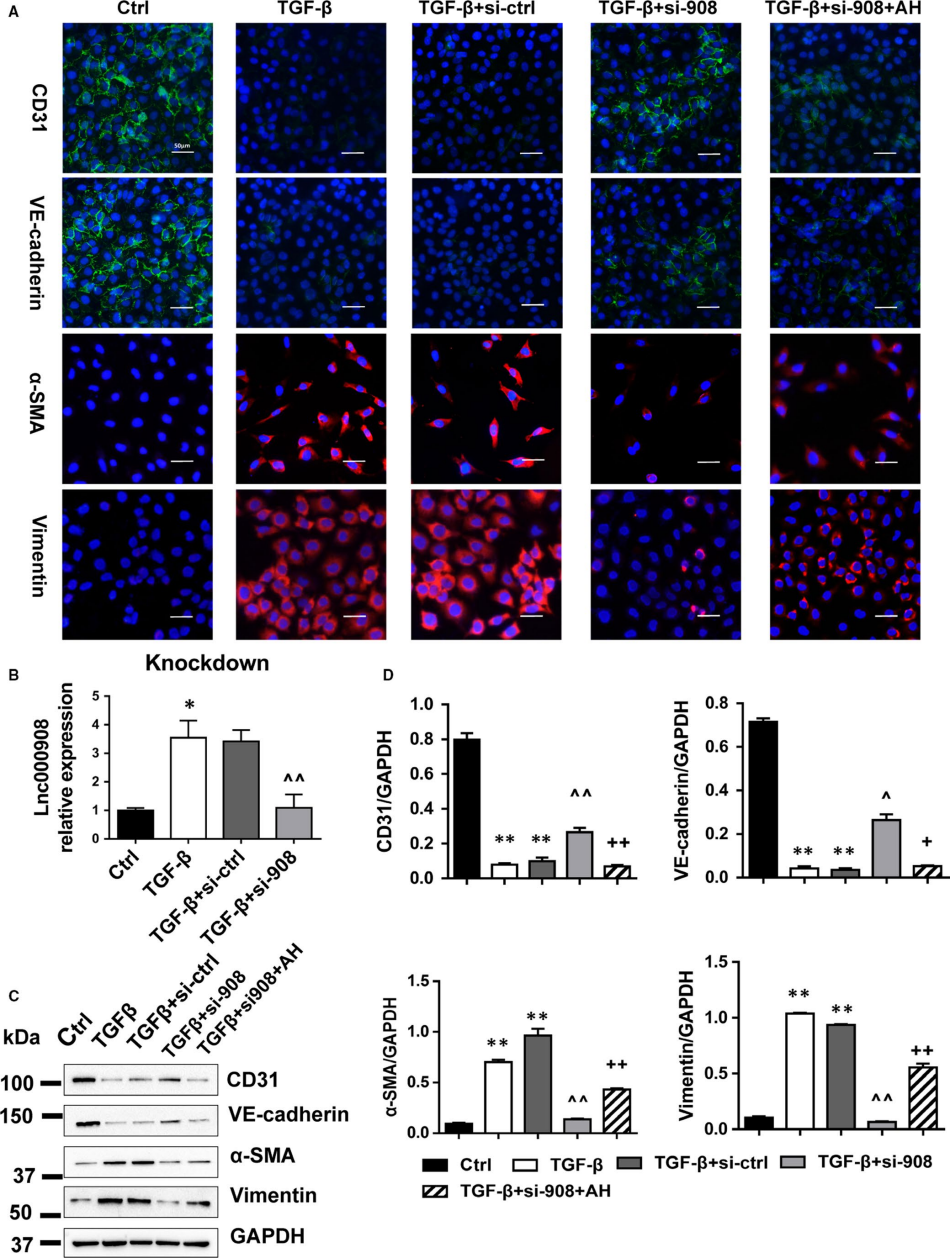
AH‐23848 reverses the anti‐EndMT effects of lnc000908 silencing. A, Immunofluorescence assay of EndMT markers. Cells were transfected with lnc000908‐targeted siRNA (si‐908) or non‐target control siRNA (si‐ctrl) for 48 h before another 24 h for TGF‐β (10 ng/mL). AH‐23848 (AH, a selective EP4 antagonist) was administrated 4 h after TGF‐β exposure. Magnification ×40. CD31 and VE‐cadherin, green; α‐SMA and Vimentin, red; nucleus were stained in blue with DAPI. B, Transfection efficiency was assessed by qRT‐PCR of lnc000908 expression, n = 4. C and D, Western blot analysis of EndMT markers, n = 5. Each experiment was repeated at least for three times. Data are presented as means ± SD. *P < 0.05, **P < 0.01 vs ctrl; ^*P* < 0.05, ^^*P* < 0.01 vs TGF‐β + si‐ctrl; +*P* < 0.05, ++*P* < 0.01 vs TGF‐β + si‐908

**Figure 6:**
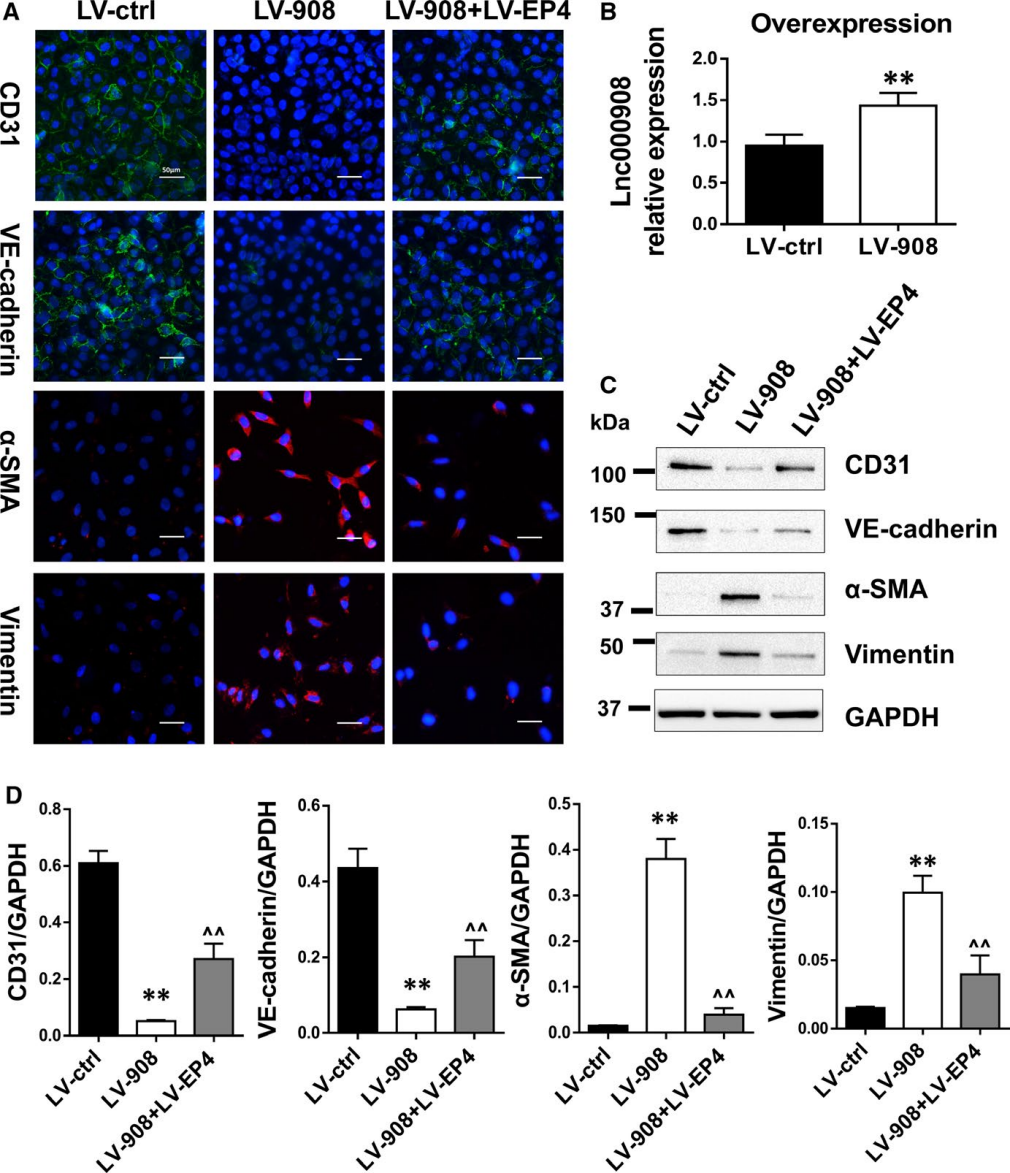
Overexpression of lnc000908 induced EndMT in primary CMECs. A, Immunofluorescence staining of EndMT markers. CMECs were pre‐incubated with lnc000908‐targeted lentivirus (LV‐908) or control lentivirus (LV‐ctrl) for 48 h and further infected with LV‐EP4 for additional 48 h. Magnification ×40. CD31 and VE‐cadherin, green; α‐SMA and Vimentin, red; nucleus were stained in blue with DAPI. B, Enhanced lnc000908 expression was demonstrated by qRT‐PCR analysis, n = 4. C and D, Western blot analysis of EndMT markers, n = 4‐5. Each experiment was repeated at least for three times. Data are presented as means ± SD. **P < 0.01 vs LV‐ctrl; ^^*P* < 0.01 vs LV‐908; CMECs, cardiac microvascular endothelial cells

Owing to the lnc000908 endothelial dominant expression in the heart, we hypothesized that the protective effects of silencing lnc000908 could be mediated by EndMT. As shown in Figure 4A,B, Iso treatment down‐regulated CD31 but increased the α‐SMA expression in the cardiac microvascular endothelium, namely enhanced EndMT, but silencing lnc000908 reversed the EndMT process. In addition, the EndMT marker expression was established by the Western blot analysis (Figure 4C). Likewise, in primary cultured CMECs, the lnc000908 knockdown also blocked TGF‐β‐induced EndMT and demonstrated by CD31, VE‐cadherin, α‐SMA and vimentin protein expression (immunofluorescence stain in Figure 5A and Western blot in Figure 5C,D). In contrast, the lnc000908 overexpression by lentivirus directly increased the α‐SMA and vimentin expression and decreased CD31 and VE‐cadherin protein by immunofluorescence staining (Figure 6A) and Western blot (Figure 6C,D). The knockdown or overexpression efficiency was assessed by the qRT‐PCR analysis of the lnc000908 expression (Figures 5B and 6B). These findings suggested that silencing lnc000908 decreased cardiac fibrosis and enhanced the cardiac pump function by inhibiting EndMT.

### 3.3 Lnc000908 promoted EndMT by inhibiting the EP4 receptor

**Figure 7:**
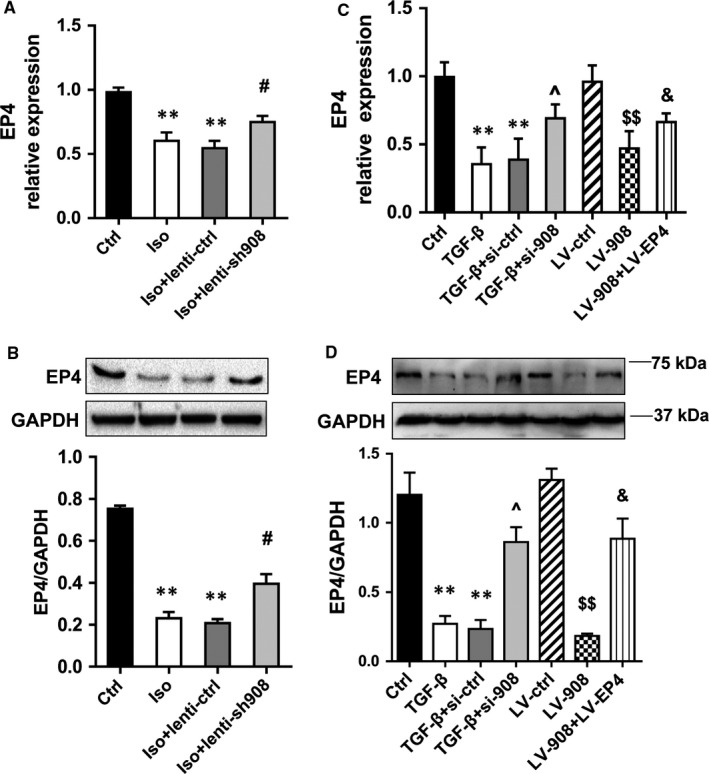
Lnc000908 regulates the expression of EP4 in vivo and in vitro. A and B, QRT‐PCR (A) and Western blot analysis (B) of EP4 expression in rats, n = 4. C and D, QRT‐PCR (C) and Western blot analysis (D) analysis of EP4 expression in primary CMECs, n = 4. Each experiment was repeated at least for three times. Data are presented as means ± SD. **P < 0.01 vs ctrl; #*P* < 0.05 vs Iso + lenti‐ctrl; ^*P* < 0.05 vs TGF‐β + si‐ctrl; $$*P* < 0.01 vs LV‐ctrl; &*P* < 0.05 vs LV‐908. CMECs, cardiac microvascular endothelial cells

We analysed the lnc000908 gene location to identify the molecular mechanism of lnc000908. The lnc000908 gene (Chr2:54944469‐54952813) was located in the upstream of its predicted target gene EP4 (Chr2:54952813‐54963448) but in the opposite transcript direction (Figure S1A), and is poorly conserved across species (Figure S1B). Thus, there is possibility that the lnc000908 might mediate EndMT by EP4. First, the qRT‐PCR and Western blot analysis revealed that both the RNA and protein expression of EP4 were markedly reduced in the fibrotic hearts of Iso‐treated rats but were up‐regulated after the lnc000908 knockdown (Figure 7A,B). In CMECs, the EP4 expression was consistent with that in the in vivo experiment (Figure 7C,D). The lnc000908 overexpression in CMECs decreased the mRNA and protein levels of EP4 (Figure 7A,B). More importantly, the cooverexpression of lnc000908 and EP4 in CMECs reversed lnc000908‐induced EndMT (Figure 7). Conversely, silencing lnc000908 inhibitory effects on EndMT were also blocked by AH‐23848 (a selective EP4 antagonist). These findings revealed that lnc000908 was directly related to EP4 and could inhibit the EP4 expression to promote EndMT in Iso‐induced cardiac fibrosis.

## 4 DISCUSSION

This study provided evidence that the lncRNA misexpression exists in the development of Iso‐induced cardiac fibrosis in rats. We identified a novel lnc000908 especially up‐regulated in the cardiac fibrogenesis. The lnc000908 knockdown reduced EndMT in vivo to improve cardiac fibrosis and heart function. In addition, the overexpression or knockdown of lnc000908 in vitro induced or suppressed EndMT, whereas down‐regulated or up‐regulated the EP4 level, respectively. Of note, these pro‐ or anti‐EndMT effects resulting from the lnc000908 intervention were reversed by the EP4 overexpression or EP4 antagonist AH‐23848. Overall, the findings demonstrated that the profibrotic action of lnc000908 was mediated by the down‐regulation of EP4, and lnc000908 inhibited the EP4 expression to promote EndMT to participate in cardiac fibrosis.

Cardiac fibrosis represents an adaptive response of the heart to various stress. After the injury, activated CFs produce and secrete soluble procollagen I and III, which are processed by metalloproteinases, cross‐linked by lysyl oxidases and hydroxylases, and finally formed into dense fibres.^1^ As the predominant matrix‐producing cells in the fibrogenesis,^24^ the proliferation of fibroblasts and its trans‐differentiation into myofibroblasts have been comprehensively investigated. Besides CFs, EndMT is a vital contributor to cardiac fibrosis and is increasingly emphasized. Endothelial cells underwent EndMT to directly expand the fibroblast pool by the loss of endothelial cell characteristics and gain of mesenchymal cell properties.^3^ In addition, diverse signalling pathways, like TGF‐β signalling, and different transcription factors, such as Twist, Snail, Slug and ZEB1/2, have been reported to participate in the EndMT process to control cell differentiation.^25^, ^26^, ^27^, ^28^, ^29^ As another member of non‐coding RNA, miRNAs molecules are around 22 nucleotides long.^30^ To date, several miRNAs, such as miR‐200a, miR‐21 and miR‐125b, have been known to inhibit EndMT by directly targeting transcription factors or inhibiting signalling pathways associated with EndMT.^31^, ^32^, ^33^

Different from the well‐documented miRNA, insights of lncRNAs into cardiac fibrosis have just started appearing. Recent studies have reported the involvement of several new lncRNAs in cardiac fibrosis.^20^, ^34^, ^35^, ^36^, ^37^ Huang et al^35^ identified 35 lncRNAs strongly related to cardiac fibrosis in the hearts of patients with ischaemic cardiomyopathy. In addition, gain‐ and loss‐of‐function experiments in CFs revealed that these lncRNAs markedly regulated the expression of the ECM synthesis gene, including Col8A1, Col3A1 and FBN1. Nonetheless, the role of the selected lncRNAs in vivo remains unsolved in this study. Wisper was another fibroblast‐enriched lncRNA that regulated the gene expression of cell proliferation, survival, extracellular matrix deposition, collagen cross‐linking and matrix stability.^36^ Reportedly, in vivo Wisper silencing markedly decreased cardiac fibrosis and improved heart failure due to myocardial infarction. The inhibition of CF‐enriched lncRNA Meg3 prevented the cardiac MMP‐2 induction, resulting in decreased cardiac fibrosis and enhanced diastolic performance.^20^ These studies suggested that lncRNA might supply novel approaches to cardiac fibrosis treatment; however, all these lncRNAs are studied in CFs. To date, no evidence of lncRNA in cardiac EndMT has been reported.

In this study, lnc000908 is markedly up‐regulated in fibrotic heart and mostly expressed in cardiac vascular endothelial cells. In addition, qRT‐PCR revealed that the lnc000908 expression was increased in primary TGF‐β‐treated CMECs. We then overexpressed lnc000908 in primary CMECs and found that the lnc000908 overexpression induced EndMT in primary CMECs. In contrast, we next performed the preventive lnc000908 depletion in primary CMECs before the induction of EndMT by TGF‐β; the results revealed that EndMT was remarkably attenuated in the lnc000908 knockdown CMECs. In addition, we established the profibrotic effect of lnc000908 in vivo. The lentivirus‐mediated lnc000908 silencing alleviated cardiac fibrosis and enhanced the impaired cardiac function in Iso‐treated rats. The reduced expression of CD31 and the increased expression of α‐SMA indicated the existence of EndMT in Iso‐treated heart, consistent with our previous work.^6^, ^7^ The lnc000908 knockdown hindered the process of EndMT in vivo. Unlike other cardiac fibrosis‐related lncRNA, lnc000908 is a novel profibrotic lncRNA that focused on EndMT, but not fibroblasts, to aggravate cardiac fibrosis and cardiac dysfunction.

EP4 is the most extensively distributed PGE2 receptor subtype in the heart^8^ and has been shown to play an antifibrotic role.^11^, ^12^ Remarkably, the coexpression analysis revealed that the predicted target gene EP4 was closely located with lnc000908, implying that there might be a correlation between them. Our findings revealed that the mRNA and protein expression of EP4 was lowered in the fibrotic heart and TGF‐β‐treated primary CMECs. In addition, the lnc000908 knockdown increased, but the forced expression of lnc000908 decreased the mRNA and protein levels of EP4 in vitro, suggesting that EP4 was the direct downstream target gene of lnc000908. In this study, we noticed that the regulation of lnc000908 on EndMT was accompanied by the opposite change of the EP4 level. We further found that the improvement in EndMT resulted from the lnc000908 knockdown was abolished by pharmacological blocking of EP4 with AH‐23848. Likewise, the lentiviral overexpression of EP4 also mitigated the increased EndMT caused by the forced expression of lnc000908. Our findings elucidated that EP4 mediates the EndMT regulation of lnc000908 in cardiac fibrosis. Overall, these findings strengthen our hypothesis that lnc000908 reduces the EP4 expression to promote EndMT, thereby facilitating the progress of cardiac fibrosis.

LncRNAs affect almost every stage of the protein‐coding gene expression through epigenetic modification, transcriptional regulation and post‐transcriptional processing.^38^, ^39^ Some lncRNAs have been reported as competing endogenous RNA (ceRNA) to regulate cardiac fibrosis at the post‐translational level; for example, lncRNA H19 functions as ceRNA to mediate the CTGF expression by sponging miR‐455 in cardiac fibrosis.^40^ A study reported that lncRNA PFL contributes to cardiac fibrosis by acting as a ceRNA of let‐7d.^37^ Some lncRNAs bind to the chromatin modifier complex or transcription factors to mediate gene activation or repression.^41^ Wang et al^42^ unveiled that lncRNA Chaer is essential for cardiac hypertrophy progress by directly interfering with PRC2 to genomic loci, thereby inhibiting histone H3 lysine 27 methylation at the promoter regions of genes. Among all lncRNAs, large intergenic RNAs (lincRNAs) exhibit a potent capability to recruit histone modifier enzymes^43^ and have been correlated with chromatin modifier complexes like PRC2.^44^ As lnc000908 is a lincRNA and affects both RNA and protein levels of EP4, we assume that the EP4 regulation probably occurs at the transcriptional stage or even before. Perhaps, lnc000908 might regulate transcription factors or methylation and histone modification to repress EP4 transcription and, thus, promote EndMT to result in cardiac fibrosis. As our current data cannot answer how lnc000908 precisely inhibits the EP4 expression, comprehensive studies are warranted to elucidate the underlying mechanism in our future work.

Few lncRNAs are highly conserved across species compared with protein‐coding genes,^45^ and we failed to find the human homologous sequences of lnc000908. Despite sequence conservation, it also contains the conservation of structure, function and expression from syntenic loci.^45^, ^46^ We will further detect lnc000908 expression in human hearts.

## 5 CONCLUSIONS

This study elucidates that lnc000908 is a crucial mediator in the rat model of cardiac fibrosis. In addition, the inhibition of lnc000908 ameliorates cardiac fibrosis and heart function by up‐regulating the EP4 level to hinder the EndMT process. Overall, our findings provide evidence that lncRNA might be a new antifibrotic target in the therapy of cardiac fibrosis.

## CONFLICT OF INTEREST

The authors confirm that there are no conflicts of interest.

## AUTHOR CONTRIBUTION

Hao Zhou and Bin Geng designed the study, offered important suggestion to manuscript writing and did manuscript final approval. Wenhua Ge, Jie Hu, Tiancheng Dong, Hui Yao and Lingzhi Chen performed in vivo and in vitro experiments. Xingxing Chen performed data analysis and wrote the paper.

## Data Availability

All data that support the findings of this study are available from the corresponding author upon reasonable request.
