# Supplementary figures and images for: Inhibition of prostaglandin E2 receptor 4 by lnc000908 to promote the endothelial‐mesenchymal transition participation in cardiac remodelling
Source: J Cell Mol Med. 2019 Jul 12;23(9):6355–67. doi: 10.1111/jcmm.14524 (PMC6714495; doi:10.1111/jcmm.14524)

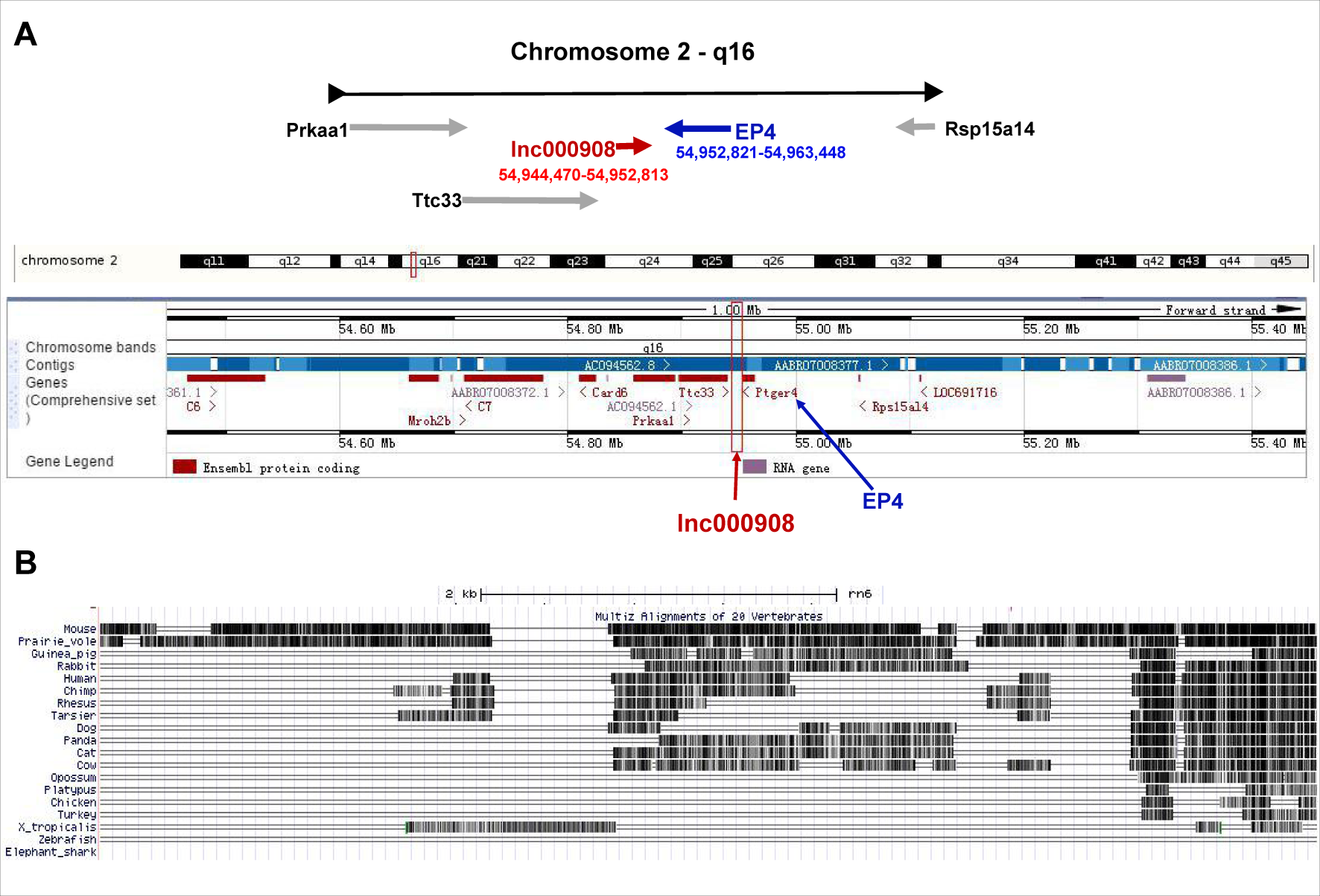

Supplement: Supplementary file 1 [file JCMM-23-6355-s001.tif]
